# Legal Underpinnings of the Great Vaccine Debate of 2025

**DOI:** 10.1017/jme.2025.51

**Published:** 2025

**Authors:** James G. Hodge

**Affiliations:** Sandra Day O’Connor College of Law, Arizona State University, Phoenix, Arizona, United States

**Keywords:** vaccine, law, policy, debate, federal

## Abstract

Multiple factors aligning in 2025 implicate challenges to vaccines as a primary public health intervention. Anti-vaccine proponents seek to recast immunization policies in promotion of perceived individual liberties. Recalibrating national vaccine approaches, however, runs counter to long-standing public health laws and policies grounded in a core truth: safe and effective vaccines save lives.

One quarter into the twenty-first century and the US finds itself — yet again — questioning the efficacy, safety, and utility of vaccines.^1^ What’s ironic underlying the “great vaccine debate of 2025” is how similar disputes were raging over a hundred years ago despite evidence of the efficacy even then of vaccines.^2^ Proof of vaccine effectiveness is now well settled. Tens of millions of deaths and immeasurable morbidity have been avoided over the last century via immunizations.^3^ That vaccine counterarguments are arising in the backdrop of the real-time development and distribution of safe and effective COVID-19 vaccines is remarkable.^4^ Millions of lives were spared from COVID-19 through inoculation.^5^

Americans are increasingly divided over vaccines as the second administration of President Donald Trump commences in January 2025. On one side, an overwhelming majority of public health officials, medical practitioners, and lawmakers and policymakers seek to preserve wide-scale vaccinations of eligible populations for largely preventable, infectious conditions.^6^ They understand the immense benefits of inoculations, trust the underlying science, appreciate how vaccine mandates help assure herd immunity, view vaccination among their civil responsibilities, and are undeterred about remote risks of adverse reactions to vaccines.

Conversely, a smaller, vocal number of Americans share an overall negative view of immunizations. Despite undeniable public health achievements of successful vaccine campaigns, “anti-vaxxers” seriously question the science justifying mass-scale inoculations.^7^ They disdain government edicts requiring them or their children to get vaccinated as a condition of societal benefits (e.g., school attendance, mass transit, travel visas, work allowances).^8^ They may depict vaccine mandates as compulsory (even though they are not — few persons are forcibly vaccinated in the US).^9^ The risks of allegedly insufficiently tested vaccines are seen as outweighing the benefits, since vaccine-preventable diseases are largely uncommon in the US.^10^ Although the relative absence of diseases like measles, polio, and whooping cough is due to decades of effective vaccine practices,^11^ some would rather forego immunizations despite clear threats to their and others’ health.

Public health authorities have ridden waves of active vaccination resistance over decades. New to the 2025 debates, however, is the potential for high-level political appointees and operatives within the Trump administration to recalibrate vaccination laws and policies in concert with well-funded anti-vaccination organizations.^12^ Multiple persons tapped by President Trump to lead key federal health agencies, notably Robert F. Kennedy, Jr. as Secretary of the Department of Health and Human Services (HHS),^13^ are highly skeptical of vaccinations. Like President Trump,^14^ they have publicly questioned the safety of and need for immunizations, reflecting the sentiments of some federal and state legislators, state agency heads, judges, and other Americans seeking to reevaluate vaccine approaches.^15^

Asdiscussed below, the possibility of a national vaccine overhaul led by the federal government entails manifold legal arguments, maneuvers, and options to obviate predictable reductions in life expectancies and rises in morbidity.

## Assessing Potential Legal Threats to Vaccination

President Trump’s return to the White House in 2025 raises the specter of profound shifts in vaccine policies. Even though his prior administration was responsible for the real-time development and production of COVID-19 vaccines in 2020 — a remarkable feat which President Trump lauded — his overall approach to vaccines has shifted.^16^ Espousing anti-science rhetoric during his 2024 presidential campaign, he questioned the efficacy of vaccines, challenged vaccine mandates, publicized the disproven link between vaccines and autism, and promised to defund schools requiring immunizations among students and staff.^17^ A driving premise among President Trump and his appointees is their perception that constitutional liberty principles support autonomous choices of Americans to get vaccinated — or not — without heavy-handed government intervention.

Diverse legal measures may arguably be invoked to effectuate short- and long-term anti-vaccine initiatives. At the federal level, calls to revisit and revise CDC national vaccine recommendations through its Advisory Committee on Immunization Practices (ACIP)^18^ present an opportunity for rapid, real-time adjustments. Highly influential ACIP recommendations are tied to specific governmental funding allocations and private sector health insurance coverage. Partial reversal or complete rejection of ACIP endorsements for specific vaccines may reshape the portfolio of inoculations available to children and adults.

Vaccines are only considered by ACIP after the Food and Drug Administration (FDA) either (1) licenses them following comprehensive approval processes,^19^ or (2) temporarily authorizes them under close reviews largely in public health emergencies (PHEs).^20^ Both of these processes — approvals or emergency authorizations — are controlled by FDA under myriad factors. Excessive data demands or delays within FDA’s bureaucracy can inhibit timely vaccine development and access. Efforts to limit FDA’s emergency use authorizations may stymie access to safe vaccines amid threats from emerging communicable diseases like H5N1 (avian flu).^21^ Direct challenges to FDA’s approval of specific vaccines, including polio,^22^ could lead to their temporary or permanent ouster from the market.^23^

Even among approved or authorized vaccines, FDA can require specific warnings on their labels.^24^ Typically, information of prospective adverse events associated with vaccines focuses on clear and present hazards which reasonable medical practitioners and patients may need to know (e.g., specific adverse outcomes for pregnant patients). Posting of information on imperceptibly small risks (e.g., a one in ten million chance of developing a treatable condition after vaccination) is atypical.

FDA determinations concerning vaccine labels, however, are fungible. Information about rare risks of vaccines appearing on FDA-approved labeling may skew public perceptions of potential harms of inoculation. Many Americans lack sufficient scientific literacy to properly assess medical risks.^25^ As retired US Supreme Court Justice Stephen Breyer espoused, individual reliance on heuristic rules of thumb for understanding risks can overstate actual harms.^26^ Research proposals advanced by Robert F. Kennedy, Jr. to investigate prospective harms of existing vaccines may suggest additional, infinitesimal risks,^27^ leading some people to avoid immunizations based on their misperceptions of risk instead of actual benefits.

Rampant misinformation underlying vaccinations and their administration already inhibits their uptake. Invoking First Amendment principles, skeptical federal officials under the Trump administration may share misleading or incomplete data regarding vaccine risks, efficacy, and mandates. Even if governmental officials do not directly spread or fund such messaging, failing to limit private sector false communications regarding vaccine harms devolved from conspiracy theories, hoaxes, or other sources is dangerous. Lack of governmental action in such instances may imply endorsement, allowing misinformation to overtake truthful data on the need for widespread vaccinations, especially among vulnerable populations (e.g., children, elderly, immunocomprised persons).^28^

In uncommon cases where approved or authorized vaccines clearly contribute to actual harms among patients, Americans can recover direct costs through federally funded programs like the National Vaccine Injury Compensation Program.^29^ Created in 1986 to help assure some recourse for affected individuals, it relies on continued Congressional support. Defunding or scaling down the program may encourage some to avoid vaccines produced by pharmaceutical companies they already distrust.

Federal purchasing powers have allowed millions of Americans seeking vaccination to actually get them for decades. Withdrawing Congressional and related health agency funding for immunization programs can derail public access. CDC’s essential Vaccines for Children Program relies annually on federal funds to assure millions of at-risk kids get vaccines approved by ACIP.^30^ Further, budget reductions for the Strategic National Stockpile (SNS) may result in supply failures when vaccines are needed most in PHEs.^31^ Resources allocated to state and local governments by other federal agencies, such as the Department of Education, could be withheld by Congress to schools adhering to existing vaccine mandates.

Tens of millions of Americans’ health plans include specific vaccine benefits extending from the Affordable Care Act (ACA) signed by President Obama in 2010.^32^ President Trump repeatedly failed to repeal and replace the ACA in his first administration.^33^ A considerably easier legislative objective in a Republican-majority House and Senate in 2025 would be to simply delete vaccine coverage in the ACA’s essential health benefits.^34^ Americans would still have a choice to get immunized, but their health plans would not be responsible for covering vaccines. Shifting costs of coverage to individuals instead of insurers would limit vaccine access nationally.

While the innumerable public health benefits of vaccines were a “*cause celebre*” among prior federal health agencies,^35^ the Trump administration may promote anti-vaccination approaches by quelling information of underlying dangers. Prior gaps in herd immunity for specific conditions often surface when CDC, Department of Agriculture (USDA), or other federal agencies investigate and uncover emerging infectious diseases (e.g., measles). Defunding or limiting federal investigators from identifying or confirming outbreaks will not stop disease spread but will inhibit public awareness.

Refusing to declare PHEs during outbreaks is another executive option. PHEs allow for real-time efforts to control rampant spread of vaccine-preventable conditions, including vaccine mandates for health care workers, liability protections for manufacturers and distributors via the PREP Act,^36^ and SNS disbursements to state and local governments.^37^ Each of these measures relies on HHS’ Secretary or other federal leaders to actually invoke these emergencies.^38^ Without a legal declaration there is technically no emergency. Absent declared emergencies, muted public health responses may be ineffective or insufficient.

Judicial interventions directly impact vaccine laws and policies too. For decades, the US Supreme Court (SCOTUS) has stood behind its federalism-based decisions allowing state and local vaccine requirements^39^ and school vaccination mandates.^40^ SCOTUS has indirectly endorsed religious exemptions to vaccines by steadfastly refusing to review lower court cases.^41^ SCOTUS did curb federal vaccine mandates set by the Occupational Safety and Health Administration (OSHA) for large employers during the COVID-19 pandemic in 2022.^42^ However, it simultaneously allowed HHS’ Centers for Medicare and Medicaid Services (CMS) to require health care providers to be vaccinated on different legal grounds.^43^

SCOTUS and lower courts may be primed to reconsider their stances amid vaccine debates. Following the Court’s 2024 administrative law decision in *Loper*, federal courts are required to forego agency deference in resolving disputes over regulations amid legislative ambiguities.^44^ Litigants are incentivized to frame vaccine-related cases as raising questions of Congressional intent. SCOTUS also faces repeated pleas for its constitutional opinion on religious exemptions from vaccine mandates under the First Amendment Free Exercise clause. The possibility of SCOTUS’ requiring religious exemptions for any vaccine mandate carries major repercussions. Numerous childhood infectious disease outbreaks occur each year in communities whose parents claim religious exemptions for their children from school vaccination mandates.^45^ The correlation is clear: as religious exemptions rise, so do infectious diseases.

## Countering Anti-vaccination Laws and Policies

Potential public health impacts of aggressive deployment of federal law and policy options pursuant to an "antivax playbook" are staggering. Vaccine approvals or authorizations may be delayed or foregone. Agency adoption of specific vaccines for national implementation could be reconsidered. New studies finding remote harms of immunizations may shape public trust. Unfettered spread of misinformation or withdrawals of funding may dissuade millions of Americans from getting vaccinated. Long-standing mandates may be relaxed or lifted altogether. Religious and other exemptions from any remaining mandates could be instituted. Prospective consumer harms arising directly from vaccines, though unusual, may find little recourse. Known (and unknown) rates of preventable infectious diseases and outbreaks may rise, resulting in excess morbidity and deaths for which lawmakers or political actors will not want to be held accountable.

While the potential tolls of anti-immunization strategies are steep, they are also avoidable through adept legal, policy, and political counterapproaches. As noted, antivax sentiments are not novel. Virtually every argument against vaccines in 2025 has surfaced before. Each faces substantial challenges under nearly 150 years of pro-vaccination public health laws and policies in the U.S. and globally. The sands of national immunization practices may be shifting, but the foundation of legal support for vaccines remains intact.

The federal government has national influence, resources, and premier responsibilities over vaccine approvals and authorizations. State and local governments, however, largely set vaccine requirements, especially concerning vulnerable populations in schools, daycares, nursing facilities, hospitals, and other settings.^46^ CDC and other agency recommendations for vaccine uptake do not automatically control state and local public health departments’ decisions to require immunizations. State legislatures and some public health agencies can adopt their own approaches apart from federal vaccine standards currently incorporated by reference into many state laws.^47^

Heavy-handed federal efforts to commandeer traditional state and local public health powers via spending limitations or enforcement mechanisms face stiff tests under principles of federalism. Public health actors on the front lines of preventable disease outbreaks may legally respond in myriad ways. Federal threats to defund schools in states requiring vaccine mandates, for example, may be met by state-based enforcement of emergency measures to limit access of unvaccinated children or staff to schools during disease outbreaks via state or local PHEs.^48^ Multiple states including California and New York limit vaccine exemptions to cases where immunocompromised persons may be at direct risk from inoculation.^49^ While some conservative states have adopted antivax laws removing mandates, allowing extensive exemptions,^50^ or inhibiting information-sharing about vaccines,^51^ none actually forbid persons from receiving vaccines (unlike some states prohibiting gender affirming care for minors).^52^

Federal agencies also do not control other key actors at the forefront of vaccine debates, specifically pediatricians, nurses, and other health professionals. Health care workers are licensed by states under their scope of practice and pursuant to standards of care guided by ethical principles centered on the best interests of patients.^53^ Questionable factors raised via federal agents over the efficacy and safety of childhood and other vaccinations may be countered by doctors responsible for their patients’ health outcomes. Credible national medical associations routinely recommend vaccinations for health care personnel and patients. Parental refusals to vaccinate their children are already a fast ticket out of many pediatricians’ offices due to the risks of harm to affected children.^54^ Vaccination is the standard of care among most health practitioners; failing to meet that standard leads to legal complications for patients and practitioners.

Medical professionals’ insistence on patient vaccinations as a condition of continued treatment may be supported by health insurers seeking to avoid massive costs over the short and long term of treating unvaccinated patients for immediate infections and later stage conditions (e.g., cancers, “long COVID”). Conversely, antivax strategies purporting to undo ACA’s required basic coverage for many vaccines may be objected to by insurers, including Medicare, Medicaid, ERISA health plans, and private insurance exchanges that collectively cover approximately 92% of the population (~305 million persons).^55^ As costs of treating insureds’ preventable infectious diseases and related conditions under antivax approaches outweigh reasonable costs of vaccines, insurers’ calls for vaccine adherence may intensify.

This presumes that vaccine costs remain reasonable. Reaching economies of scale through federal purchasing power of vaccines, including via Medicare, is key, but is not wholly dispositive. State and local governments, private insurers, and health providers purchase large lots of vaccines. Antivax legal interventions cutting into the $30 billion annual market for vaccines,^56^ seeking to remove FDA-approved products from the market, skewing public perceptions of their safety, or diminishing company liability protections may be fiercely opposed by lobbyists of large pharmaceutical companies facing prospective lost profits.

Paramount political or economic resistance to anti-vax policies is undergirded by existing laws geared toward vaccine promotion, not diminution. As noted, state police and *parens patriae* powers empower governments to require vaccines as a condition of school attendance, employment, or other settings.^57^ Sanctions may arise under state medical licensure laws against health care workers that falsely claim vaccines are unsafe.^58^ The Americans with Disabilities Act and corresponding state disability laws protect persons who cannot be vaccinated for medical reasons from discrimination.^59^

Emergency protections from unwarranted liability claims against vaccine manufacturers are steadfast. PREP Act protections for COVID-19 vaccine makers initiated during President Trump’s first administration were recently extended by outgoing HHS Secretary Becerra until *December 31, 2029.* ^60^ Attempts to pull already FDA-approved vaccines from consumer access require definitive proof of safety risks, which can take years.^61^ Unsupported withdrawals of approved vaccinations may be countered by industry lawsuits centered on regulatory takings and other fronts.

Subject to rare exceptions noted above, SCOTUS and most lower courts regularly endorse efficacious vaccination requirements. Existing First Amendment protections do not require religious exemptions nor support the spread of false or misleading information about vaccinations via governmental sources. Individuals sharing patently false information about vaccinations may face criminal or civil libel, defamation, or fraud charges.^62^ Commercial messages grounded in misinformation enjoy no First Amendment protection and can be shut down via government.^63^ Importantly, no constitutionally sound law can stop the sharing of truthful information via public or private sectors about the benefits of vaccines. Public health education campaigns can be an effective antidote to misinformation spread via public or private officials.

While CDC or other federal agencies may decline to investigate infectious disease outbreaks, state and local governments are legally empowered to conduct their own surveillance and epidemiologic investigations. States facing appreciable risks of harms to individuals or populations from infectious diseases may also declare their own PHEs and respond accordingly.^64^ Persons responsible for intentionally spreading infectious diseases via bioterrorism or other means can be criminally or civilly liable, although proof can be hard to ascertain. Parents refusing to vaccinate their children may be charged with neglect in some jurisdictions.^65^

At the epicenter of the great vaccine debate of 2025 is a collision of two dynamics: (1) the politicization of public health in promotion of perceived liberties and (2) governmental responsibilities to promote communal health. How vaccine laws and policies may change remains undetermined with the health of millions of Americans at stake. Adept applications of existing legal foundations promoting vaccination are key to assuring the preservation of communal health in the everlasting battle over preventable infectious diseases.
